# Stable single-mode operation of distributed feedback quantum cascade laser under high current via a grating reflector

**DOI:** 10.1515/nanoph-2023-0077

**Published:** 2023-03-28

**Authors:** Fan Ye, Fengmin Cheng, Zhiwei Jia, Jinchuan Zhang, Ning Zhuo, Fengqi Liu, Youdou Zheng, Yi Shi

**Affiliations:** School of Electronic Science and Engineering Nanjing University, Nanjing, Jiangsu 210093, People’s Republic of China; Key Laboratory of Semiconductor Materials Science, Chinese Academy of Sciences, Institute of Semiconductors, Beijing 100083, People’s Republic of China

**Keywords:** DFB QCL, grating reflector, light distribution, single-mode stability

## Abstract

We report on an index-coupled distributed feedback (DFB) quantum cascade lasers (QCLs) of high single-mode stability by using of a grating reflector (GR) to reflect effectively light of a specific wavelength. Fabrications of the QCLs were performed with *λ*∕4 equivalent phase shift (EPS) or sampled Moiré grating (SMG) structure, which regulates the light intensity distribution in the cavity and coupling strength. Both EPS-GR and SMG-GR QCLs demonstrate a high emission wavelength precision and a side-mode-suppression-ratio (SMSR) of 24.5 dB at the injection current of 5 A and the pulse width of 5 μs, offering considerable output power even at the edge of the gain spectrum. Furthermore, we investigated the arrays of EPS-GR and SMG-GR QCLs with lower threshold current and higher output power by introducing semi-insulated InP (Fe) ranging from 8.25 to 8.67 μm and 8.16–8.63 μm, respectively. The present QCLs via a GR are prospective for applications requiring single-mode stability and wide tunability.

## 1 Introduction

Semiconductor quantum cascade lasers (QCLs) based on resonant tunneling and optical transitions within the conduction band of a multi-quantum-well structure, are one of the most promising light sources from the mid-infrared to terahertz wavelength range [1–7]. QCLs have various applications, including gas sensing [8], high-resolution spectroscopy [9], industrial process monitoring [10] and free space communication [11]. For practical reasons, stable single-mode operation is desired to meet the demands of most applications. In this regard, the distributed feedback (DFB) laser has been studied to achieve a side-mode suppression ratio (SMSR) above 30 dB with proper design of uniform or sampled grating [12–16], simultaneously attracting wide attention for its compactness, simple fabrication, and low cost. Metal grating also plays a significant role in single-mode DFB QCLs due to its considerable beam quality factor [17, 18], and epitaxial regrowth is not required. As a shortcoming, however, the emission wavelength precision is quite difficult to guarantee due to the dispersion in the periodic structures. In conventional index-coupled DFB QCLs, propagation is forbidden in the frequencies of the stopband, which is at the center of the Bragg wavelength with two band edges on each side [19, 20]. The same amount of loss for the two band edge modes cannot ensure true single-mode operation; that is, mode hopping always occurs. In most cases, anti-reflection and high-reflection (AR/HR) coatings are applied on front and rear facets to reflect specific wavelength light and amplify the loss difference of the two modes to obtain stable single-mode operation without mode hopping [21]. However, an unavoidable random grating phase at the HR facet still leads to degradation of the SMSR and variation of the lasing wavelength [22]. In addition, this kind of setup can only achieve single-mode operation near the threshold current (*I*_th_) and at a small pulse width, suffering from severe broadening of the emission spectrum and a decrease in the output power when a large current or pulse width is applied [22, 23]. These issues hinder practical application of DFB QCLs because realization of a stable current in the external environment and manufacture of small pulse width power sources are quite complex and expensive.

With respect to the critical issues, the DFB QCL integrated with a grating reflector (GR) by AR/HR coatings is proposed, which reflects effectively light of a specific wavelength. Additionally, a *λ*∕4 equivalent phase shift (EPS) and a sampled Moiré grating (SMG) structure [24] are also individually applied. By optimizing the period of each sampled grating [25], the lasing wavelength of the DFB QCLs can be precisely controlled and a high SMSR obtained. Moreover, high-power DFB QCLs always have a longer cavity length (*L* ∼ 4 mm), corresponding to an excessive coupling strength (*кL* >> 1), which results in concentration of the light field in the center of the cavity. In the EPS-GR and SMG-GR structures, a weaker *к* is achieved by designing an appropriate duty cycle of the sampled grating to obtain a suitable coupling strength (*кL* ∼ 1) [26]. Therefore, considerably high output power is also obtained in this case, which is available for most applications. Furthermore, the EPS-GR and SMG-GR structures can be manufactured by only one holographic exposure combined with micrometer-scale photolithography, avoiding the need for electron beam lithography (EBL), which is time consuming and expensive.

**Figure 1:**
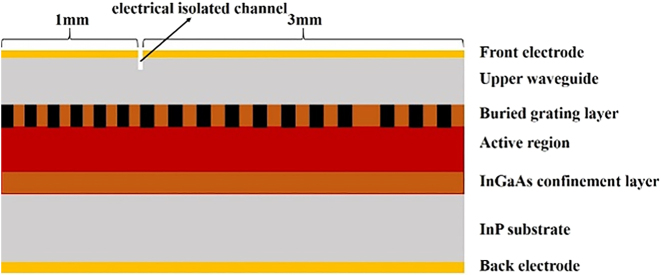
The lateral view of the EPS/SMG QCL integrated with a GR section.

In this work, we designed and fabricated QCLs employing a *λ*∕4 EPS-GR or SMG-GR structure. The EPS and SMG sections are both 3 mm long, and the GR section is approximately 1 mm long with a 50-μm-wide electrically isolated channel. A schematic of the QCLs is shown in Figure 1; the lasers achieved accurate lasing wavelengths and stable single-mode emission with swift tuning ability. All six *λ*∕4 EPS-GR QCLs and six SMG-GR QCLs can achieve an SMSR over 30 dB around the threshold current (∼1.2*I*_th_) and maintain considerable output power when the lasing wavelength is at the edge of the gain spectrum. Stable single-mode emission can still be obtained even under extremely high injection current and large pulse width. The EPS-GR and SMG-GR structures were further extended to QCL arrays, which both contain 8 lasers spacing 200 μm from the adjacent, covering emission wavelength from 8.25 to 8.67 μm and 8.16–8.63 μm, respectively. The EPS, SMG sections of lasers in arrays are both 1.8 mm and the GR section is 0.7 mm with a 50-μm-wide electrically isolated channel in the middle. Moreover, the QCLs in the arrays have better threshold current and output power, which is promising for applications requiring stable single-mode operation and high output power.

## 2 Fabrication

As shown in Figure 1, the active region of a QCL was grown on an n-doped (Si, 3 × 10^17^ cm^−3^) InP film substrate by solid-source molecular beam epitaxy. The sequence of the epitaxial layeris as follows: 3.5 μm doped lower InP cladding layer (Si, 5 × 10^16^ cm^−3^), 0.5 μm lower InGaAs layer (Si, 2 × 10^16^ cm^−3^), In_0.53_Ga_0.47_As/In_0.52_Al_0.48_As QCL active region, 0.5 μm upper InGaAs layer (Si, 2 × 10^16^ cm^−3^), 3.5 μm doped upper InP cladding layer (Si, 5 × 10^16^ cm^−3^), 0.2 μm gradually doped InP layer (Si, from 1 × 10^17^ cm^−3^ to 5 × 10^17^ cm^−3^), and 0.6 μm highly doped InP contact layer (Si, 6 × 10^18^ cm^−3^). The active region based on single-phonon continuum depopulation consists of 35 periods. The layer sequence in one period (in angstrom) from the injection barrier is as follows: **3.9**/1.3/**0.95**/5.1/**0.85**/5.0/**0.95**/4.6/**1.6**/3.5/**2.2**/2.9/**1.8**/2.7/**1.9**/2.6/ **2.0**/2.4/**2.5**/2.45/**3.1**/2.3 [27] where the bold represents barriers and the underlined represents layers with Si doped to a concentration of 1.1 × 10^11^ cm^−2^. To fabricate the buried grating, the top cladding was removed down to the upper InGaAs layer. Assuming an effective refractive index (*n*_eff_) of 3.18, the basic Bragg grating with period Λ_0_ = 1.446 μm (duty cycle *ε* = 50%) was defined by holographic exposure, followed by one optical photolithography process to form the sampled grating (duty cycle *σ* = 50%) pattern. Then, it was transferred by wet chemical etching to a depth of approximately 120 nm to form the sampled grating.

Following implementation of the sampled grating and regrowth of the InP top cladding, ridges of 10 μm were defined on the wafer by etching the surrounding area. Ridges were then passivated by a 450-nm-thick SiO_2_ layer by plasma-enhanced chemical vapor deposition (PECVD) for insulation. Then, the junctions of the EPS/SMG and GR sections in the InP top cladding were dry etched to a depth of more than 1 μm to form 50-μm-wide electrically isolated channels. Next, 3-μm-wide electrical injection windows were opened on the ridges, and the front electrodes were provided by a Ti/Au (40/250 nm) layer deposited by electron beam evaporation. Thereafter, an additional 5-μm-thick gold layer was subsequently electroplated to further improve heat dissipation. After the wafer was thinned down to 120 μm and back electrodes were formed with Ge/Au/Ni/Au, the laser bars were cleaved to a length of 4 mm. Subsequently, AR coatings of Al_2_O_3_∕Ge (700/70 nm), corresponding to a reflectivity of 1% at a wavelength of 8.3 μm, were deposited on both the rear facet and the front facet. Finally, the laser bars were mounted epi-side down on AlN heat sinks with indium solder and wire bonded.

## 3 Principle, simulation and results

### 3.1 EPS-GR

**Figure 2:**
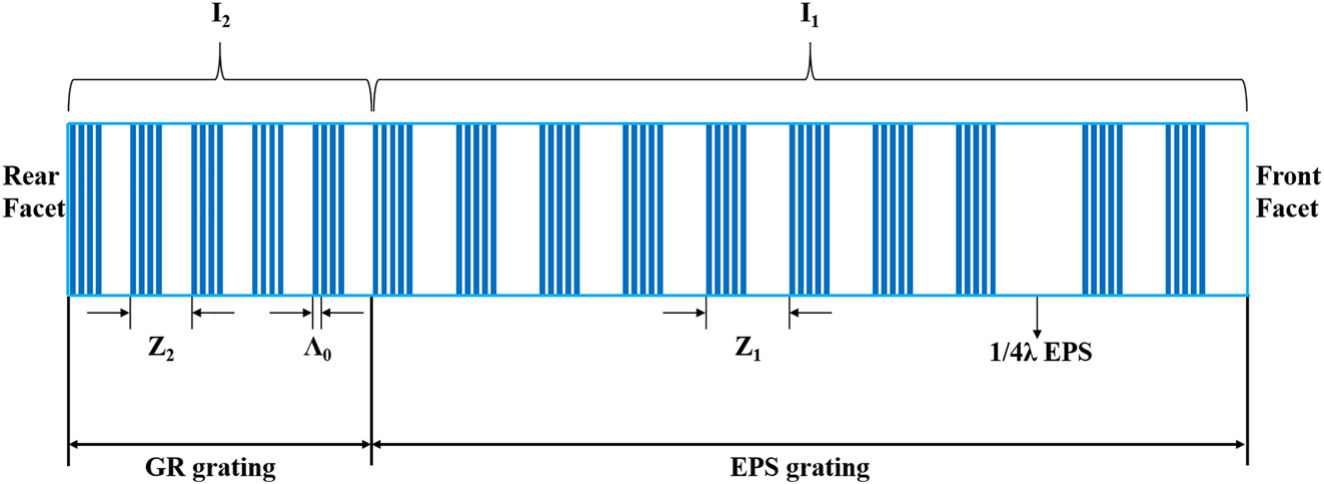
Top view of the EPS-GR grating structure.

**Figure 3:**
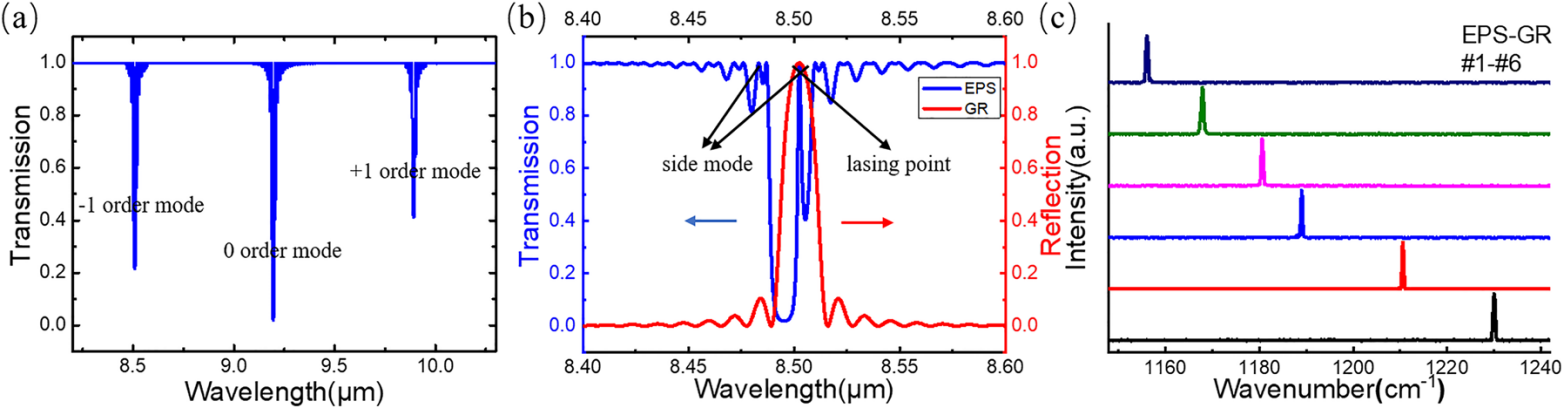
Simulated and measured emitting spectra of EPS-GR QCLs. (a) The simulated transmission of uniform sampled grating with *λ*∕4 EPS. (b) The simulated transmission of detailed −1st-order mode of *λ*∕4 EPS and reflection of the GR section. (c) The spectra of EPS-GR Laser-1 to Laser-6 measured under 1.2*I*_th_ at a duty cycle of 1% (20 kHz, 0.5 μs) at RT.

**Figure 4:**
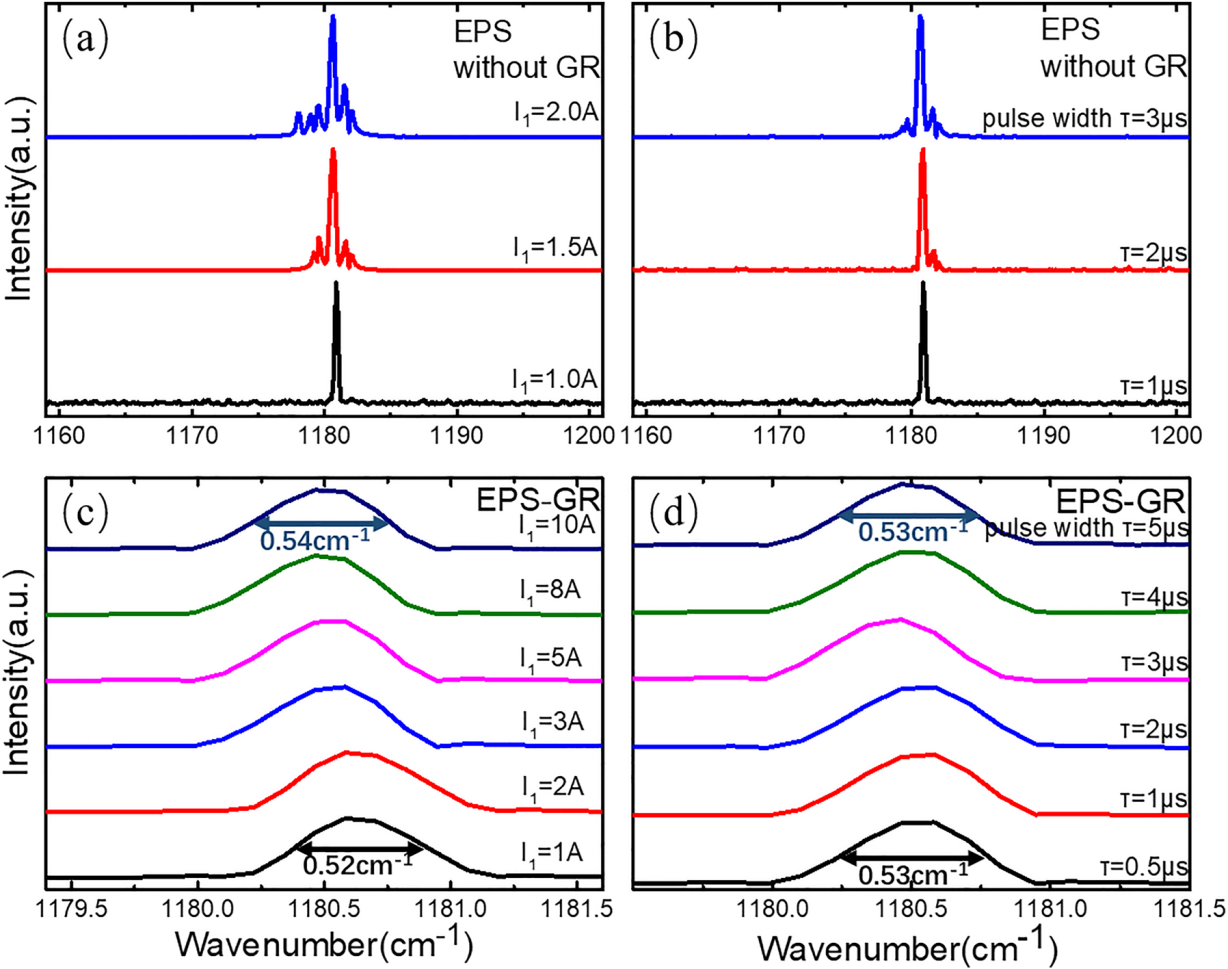
The measured spectra of the EPS (a) under different injection currents *I*_1_ from 1.0 (1.2*I*_th_) to 2.0 A with a step of 0.5 A without GR, (b) at different pulse width from 1 to 3 μs with a step of 1 μs when *I*_1_ = 1.0 A without GR, (c) under different injection currents *I*_1_ from 1 to 10 A with GR, FWHM from 0.52 to 0.54 cm^−1^, and (d) at different pulse width from 1 to 5 μs with a step of 1 μs when *I*_1_ = 5 A with GR. All measurements were implemented at the duty cycle of 1% at RT.

*λ*∕4 EPS QCLs integrated with a GR were fabricated by one holographic exposure combined with micrometer-scale lithography [28]. The EPS-GR structure is shown in Figure 2, and the basic principle of the EPS grating is expressed as:

(1) $$\lambda_{0}=2n_{\text{eff}}\Lambda_{0},$$

(2) $$\Delta \lambda =\lambda_{0}-\lambda_{1}=\lambda_{0}^{2}/2n_{\text{eff}}Z_{1},$$

where the basic Bragg grating period is Λ_0_ = 1.446 μm (duty cycle *ε* = 50%), *λ*_0_ is the Bragg wavelength of the basic grating, *n*_eff_ is the effective index of the waveguide, the sampled grating period of the EPS section is *Z*_1_. To locate lasing point in the center while side mode outside the of stop-band of GR section [25], the basic principle of the GR grating can be written as:

(3) $$\Lambda_{-1}=Z_{2}\Lambda_{0}/\left(Z_{2}+\Lambda_{0}\right),$$

(4) $$\lambda_{-1}=2n_{\text{eff}}\Lambda_{-1},$$

where the sampled grating period of the GR section is *Z*_2_, the equivalent −1st mode period is Λ_−1_, and *λ*_−1_ is the −1st^-^order wavelength. Pulse current *I*_1_ is applied in the EPS section to mainly control the emission and light intensity of lasers, while direct current *I*_2_ (∼250 mA) is applied in the GR section to compensate for the loss in this section. Figure 3(a) shows the transmission spectrum of the uniform sampled grating with the *λ*∕4 EPS structure. The odd modes generate a π phase shift after the *λ*∕4 EPS structure is employed, which breaks the stopband of the odd-order modes and introduces defect modes into the center of it. Therefore, the defect mode of the −1st order mode becomes the lasing mode due to its lower threshold compared to the +1st-order mode. Figure 3(b) shows the simulated transmission and reflection spectra of the EPS structure and GR based on the transfer matrix. The results indicate that the lasing point of the *λ*∕4 EPS structure is located at the stop-band center of the GR section, which benefits better single-mode operation. We fabricated six EPS-GR QCLs of different lasing wavelength ranging from 8.12 to 8.68 μm. Lasing spectra were measured using a Fourier transform infrared (FTIR) spectrometer under 1.2 times the threshold current (∼1.2*I*_th_) at a duty cycle of 1% (20 kHz, 0.5 μs) at room temperature (RT), as demonstrated in Figure 3(c), showing perfect single-mode performance with SMSRs all over 30 dB. The *Z*_1_ and *Z*_2_ of each laser are listed in Table 1, and the *λ*∕4 EPS section was inserted 1 mm from the front facet to obtain higher output power [25]. The emission wavelengths of all 6 EPS-GR QCLs fit well with the calculated wavelengths, which proves the emission wavelength precision of this structure. This kind of advantage can be useful for applications requiring high wavelength precision, such as wavelength division multiplexing and gas sensing. Furthermore, the peak output power of the lasers does not decline very much even when the lasing wavelength approaches the edge of the gain spectrum (∼8.12 μm). Conventional DFB QCLs often suffer severe output power degradation or cannot lase due to insufficient gain at the edge of the gain spectrum. Figure 4 shows the measured spectra of EPS-GR laser-4 working under different currents *I*_1_ in pulse mode. In conventional DFB QCLs, mode hopping and broadening occur due to the chirp effect when the laser operates under large injection current and pulse width in pulse mode. Figure 4(a) and (b) shows the lasing spectra of laser-4 without the GR section under different injection currents *I*_1_ from 1.0 (1.2*I*_th_) to 2.0 A with a step of 0.5 A at a duty cycle of 1% and under different pulse widths from 1 to 3 μs with a step of 1 μs when *I*_1_ = 1.0 A at RT. Figure 4(c) and (d) shows the lasing spectra of laser-4 for constant current *I*_2_ = 250 mA in continuous wave (CW) mode under different injection currents *I*_1_ from 1 to 10 A at a duty cycle of 1% and under different pulse widths from 1 to 5 μs with a step of 1 μs when *I*_1_ = 5 A at RT. The results indicate that the laser can only achieve single-mode operation near *I*_th_ and at a small pulse width without the GR section working and suffers from severe broadening with increasing injection current and pulse width. In contrast, the spectral linewidth slightly increases from 0.52 to 0.54 cm^−1^ with current and keeps constant (0.53 cm^−1^) with pulse width when the GR section is working. The lasers maintain considerable SMSR even under extremely high current and pulse width, which proves the function of the GR section. This clearly demonstrates that the EPS-GR QCLs exhibit excellent single-mode performance under large current and pulse width, attributed to the dual wavelength selection of two section. With advantages of steady single-mode ability and low power consumption, they can be put into use in gas sensing and industrial process monitoring, which always involve changeable external conditions. In addition, the production of a small-pulsed current power source is often costly and time consuming, and this stable single-mode device is quite reliable for ordinary power sources.

**Table 1:** The parameters for the EPS and GR sections.

Laser number	Sampling period *Z*_1_ (µm)	Sampling period *Z*_2_ (µm)	Calculated wavelength (µm)	Measured wavelength (µm)	Measured power (mW)
Laser-1	12.3	10.9	8.118	8.123	66
Laser-2	14.2	12.7	8.263	8.260	70
Laser-3	16.8	15.4	8.408	8.410	72
Laser-4	18.2	16.8	8.469	8.471	77
Laser-5	20.8	19.3	8.560	8.562	76
Laser-6	25.6	24.1	8.680	8.681	78

### 3.2 SMG-GR

**Figure 5:**
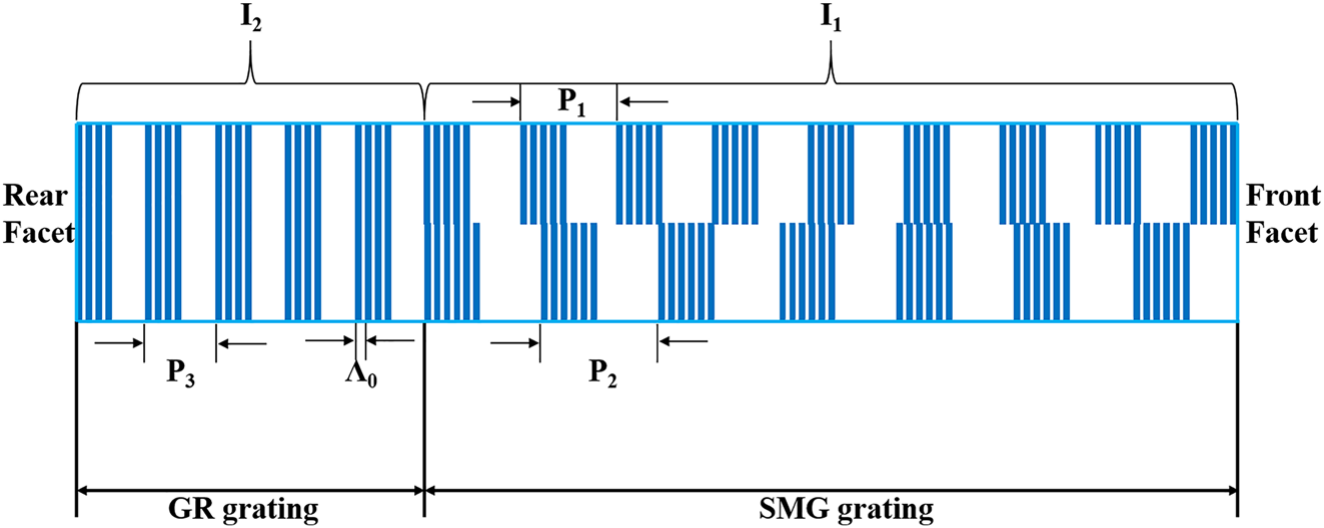
Top view of the SMG-GR grating structure.

The SMG structure is another promising structure employed in DFB lasers, as shown in Figure 5. The lasers can guarantee single-mode emission, improve the output power extraction, and suppress spatial hole burning (SHB) since the Moiré grating can induce grating apodization and a phase shift. Generally, this kind of apodization with a half-period cosine profile is almost impossible to realize by directly changing the index modulation [29]. Therefore, the SMG-GR structure can provide novel methods via flexible design to meet the requirements of practical applications for DFB QCLs. The fabrication of SMG-GR QCLs involves one conventional holographic exposure and optical photolithography. There are two adjacent sampled gratings with slightly different periods of *P*_1_ and *P*_2_ in the SMG section. *φ*_*1*_ and *φ*_*2*_ are the initial phases of the two sampled gratings, and the initial phase difference (*φ*_*1*_ − *φ*_*2*_) is set to π at the front facet to obtain the highest output power [25]. The sampled grating period is *P*_3_ in the GR section. The equivalent Bragg periods Λ_1_, Λ_2_, and Λ_3_ of the gratings of periods *P*_1_, *P*_2_, and *P*_3_ can be expressed as:

(5) $$\left\{\begin{array}{c} \Lambda_{1}=P_{1}\Lambda_{0}/\left(P_{1}+\Lambda_{0}\right),\\ \Lambda_{2}=P_{2}\Lambda_{0}/\left(P_{2}+\Lambda_{0}\right),\\ \Lambda_{3}=P_{3}\Lambda_{0}/\left(P_{3}+\Lambda_{0}\right). \end{array}\right.$$

The index modulation of the SMG can be written as:

(6) $$\begin{array}{ll} \Delta n\left(z\right) & =\frac{1}{2}\Delta n_{s}\left[\frac{1}{2}\exp\left(i\left(\frac{2\pi z}{\Lambda_{1}}+\varphi_{1}\right)\right)\right.\\ & \;\left.+\frac{1}{2}\exp\left(i\left(\frac{2\pi z}{\Lambda_{2}}+\varphi_{2}\right)\right)\right]+c\cdot c\cdot , \end{array}$$

and it can be further expressed as:

(7) $$\begin{array}{ll} \Delta n\left(z\right) & =\frac{1}{4}\Delta n_{s}\left[\frac{1}{2}\exp\left(i\left(\frac{2\pi z}{\Lambda_{s}}+\frac{\varphi_{1}+\varphi_{2}}{2}\right)\right)\right.\\ & \;\left.\times \;\exp\left(i\left(\frac{2\pi z}{\Lambda_{c}}+\frac{\varphi_{2}-\varphi_{1}}{2}\right)\right)\right.\\ & \;\left.+\;\exp\left(-i\left(\frac{2\pi z}{\Lambda_{c}}+\frac{\varphi_{2}-\varphi_{1}}{2}\right)\right)\right]+c\cdot c. \end{array}$$

where Λ_*C*_ and Λ_*S*_ can be written as:

(8) $$\Lambda_{C}=\Lambda_{1}\Lambda_{2}/\left(\Lambda_{2}-\Lambda_{1}\right),$$

(9) $$\Lambda_{S}=\Lambda_{1}\Lambda_{2}/\left(\Lambda_{2}+\Lambda_{1}\right).$$

Λ_*C*_ is much larger than Λ_*S*_ since Λ_1_ is quite similar to Λ_2_, which means that there is a rapidly varying component with a period of Λ_*S*_ and a slowly varying envelope with a period of Λ_*C*_ in the SMG section. Therefore, the integrated period of the SMG section can be recognized as Λ_*S*_, and the −1st-order wavelength can be written as:

(10) $$\lambda_{-1}=2n_{\text{eff}}\Lambda_{S}.$$

We then set the equivalent Bragg period Λ_3_ equal to the emission wavelength to make the −1st-order mode reflection higher than that of the other side modes, which can be expressed as:

(11) $$\Lambda_{3}=\Lambda_{S}.$$

Therefore, *P*_3_ can also be expressed as:

(12) $$P_{3}=2P_{1}P_{2}/\left(P_{1}+P_{2}\right).$$

**Figure 6:**
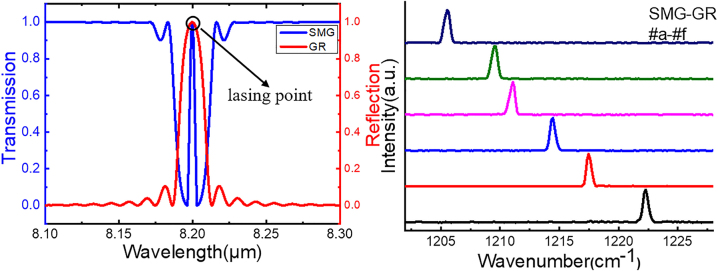
Simulated and measured emitting spectra of SMG-GR QCLs. (a) The simulated transmission of SMG and reflection of the GR section. (b) The spectra of SMG-GR Laser-a to Laser-f measured under 1.2*I*_th_ at a duty cycle of 1% (20 kHz, 0.5 μs) at RT.

Figure 6(a) shows the simulated transmission and reflection spectra of the SMG and GR based on the transfer matrix. The results demonstrate that the lasing point of the SMG is also located at the stop-band center of the GR section, and the side modes are less transmitted, which contributes to higher output power compared to the EPS-GR structure. Six SMG-GR QCLs of different periods *P*_1_, *P*_2_, and *P*_3_ were fabricated, covering the wavelength range from 8.18 to 8.30 μm. Figure 6(b) shows the lasing spectra measured under 1.2 times the threshold current (∼1.2*I*_th_) at a duty cycle of 1% (20 kHz, 0.5 μs) at RT. The *P*_1_, Λ_3_ = Λ_*S*_, *P*_2_, and *P*_3_ of each laser are listed in Table 2, and the lasing wavelengths of all 6 SMG-GR QCLs are quite close to the designed wavelengths, proving the emission accuracy of the SMG-GR structure. The average measured peak power is approximately 50% greater than that of the EPS-GR structure, showing a higher concentration at the lasing point of SMG transmission.

**Table 2:** The parameters for the SMG and GR sections.

Laser number	Sampling period *P*_1_ (µm)	Sampling period *P*_2_ (μm)	Sampling period *P*_3_ (µm)	Calculated wavelength (µm)	Measured wavelength (µm)	Measured power (mW)
Laser-a	11.5	11.7	11.6	8.180	8.181	103
Laser-b	11.9	12.1	12.0	8.211	8.214	105
Laser-c	12.3	12.5	12.4	8.240	8.234	110
Laser-d	12.6	12.8	12.7	8.260	8.257	108
Laser-e	12.8	13.0	12.9	8.273	8.268	115
Laser-f	13.2	13.4	13.3	8.298	8.295	124

**Figure 7:**
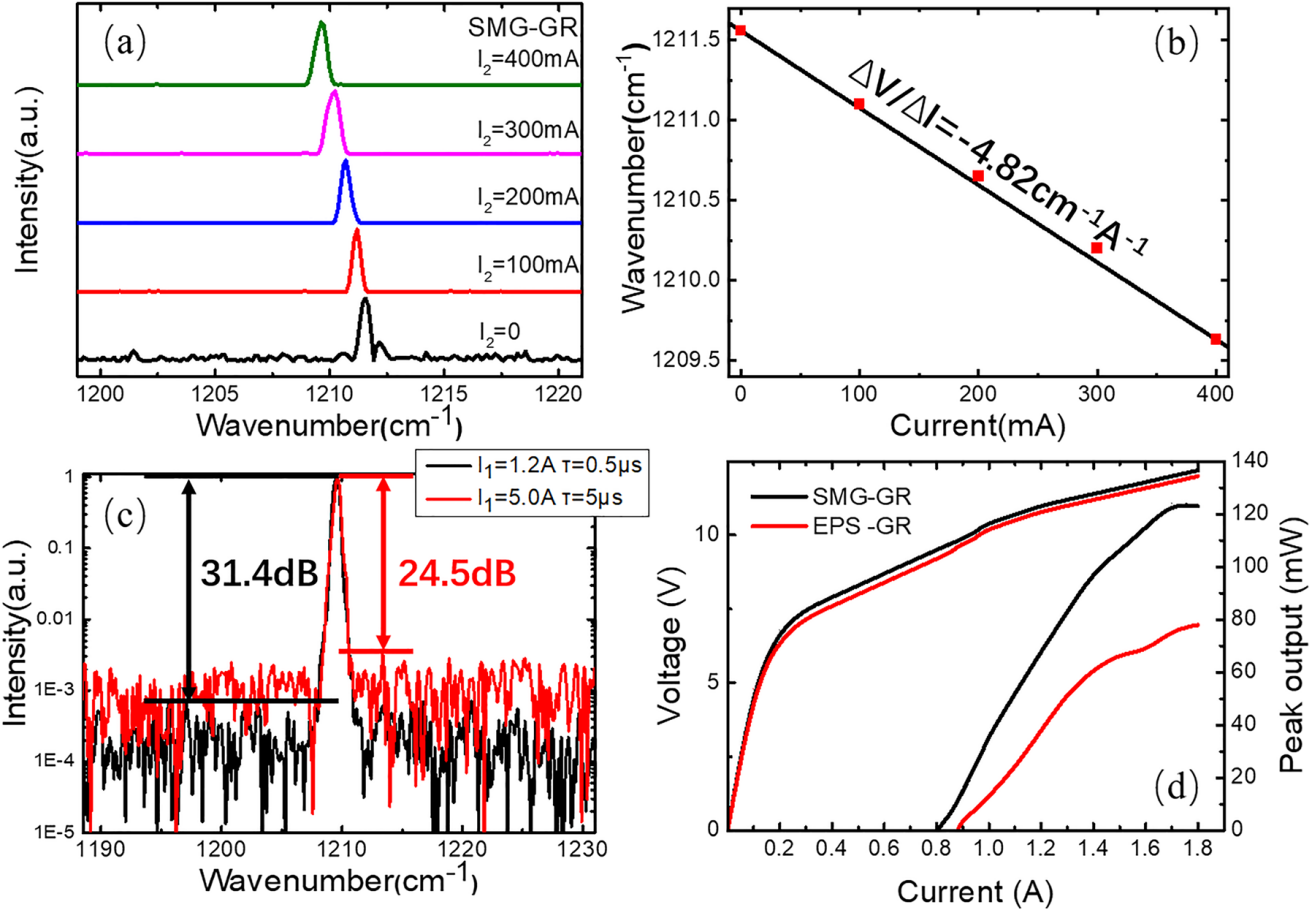
Single-mode operation and output power under different bias of SMG-GR QCL. (a) The measured spectra of the SMG-GR QCL under different injection currents *I*_2_ from 0 to 400 mA with a step of 100 mA at 1.5*I*_1_, (b) the linear tuning characteristics of the wavenumber with current, (c) the emission spectra of the SMG-GR when *I*_1_ = 1.2 A, *τ* = 0.5 μs and *I*_1_ = 5.0 A, *τ* = 5 μs, and (d) the *P*–*I*–*V* characteristics of SMG-GR and EPS-GR. All measurements were implemented at the duty cycle of 1% at RT.

Figure 7(a) shows the lasing spectra of laser-d when the constant pulse current *I*_1_ = 1.2 A (∼1.5*I*_th_) under different CW injection currents *I*_2_ from 0 to 400 mA with a step of 100 mA. The results indicate that the laser cannot work in perfect single-mode operation under 1.5*I*_th_ without the GR section working and gives better performance under larger *I*_2_ within our measuring range. The GR section still proves to be a good compromise. The center wavenumber is 1211.559 cm^−1^ when *I*_2_ = 0 and shifts to 1209.631 cm^−1^ when *I*_2_ = 400 mA, with a current tuning coefficient Δ*ν*/Δ*I* = −4.82 cm^−1^ A^−1^, as illustrated in Figure 7(b). The SMG-GR QCL exhibits a perfect linear tuning characteristic of the lasing frequency with current, which means that no mode hopping occurs when *I*_2_ is from 0 to 400 mA. This tuning is based on current, while conventional DFB QCLs can only achieve temperature tuning in pulse mode. In practical applications, changing the current is much more feasible and faster than altering the temperature, especially when the laser is operated outside. The QCL can sustain a larger CW *I*_2_ by appropriately increasing the size of the GR section, therefore further improving the current tuning range. Figure 7(c) shows the spectra measured under different *I*_1_ and pulse widths when the constant CW current *I*_2_ = 400 mA. The SMSR is 31.4 dB when *I*_1_ = 1.2 A and *τ* = 0.5 μs and is maintained at 24.5 dB when *I*_1_ = 5.0 A and *τ* = 5 μs, at which conventional DFB QCLs cannot even work. As shown in Figure 7(d), the output power of SMG-GR is much larger, since its function of light distribution and more intensity is concentrated in lasing point [25].

### 3.3 EPS-GR and SMG-GR arrays

**Figure 8:**
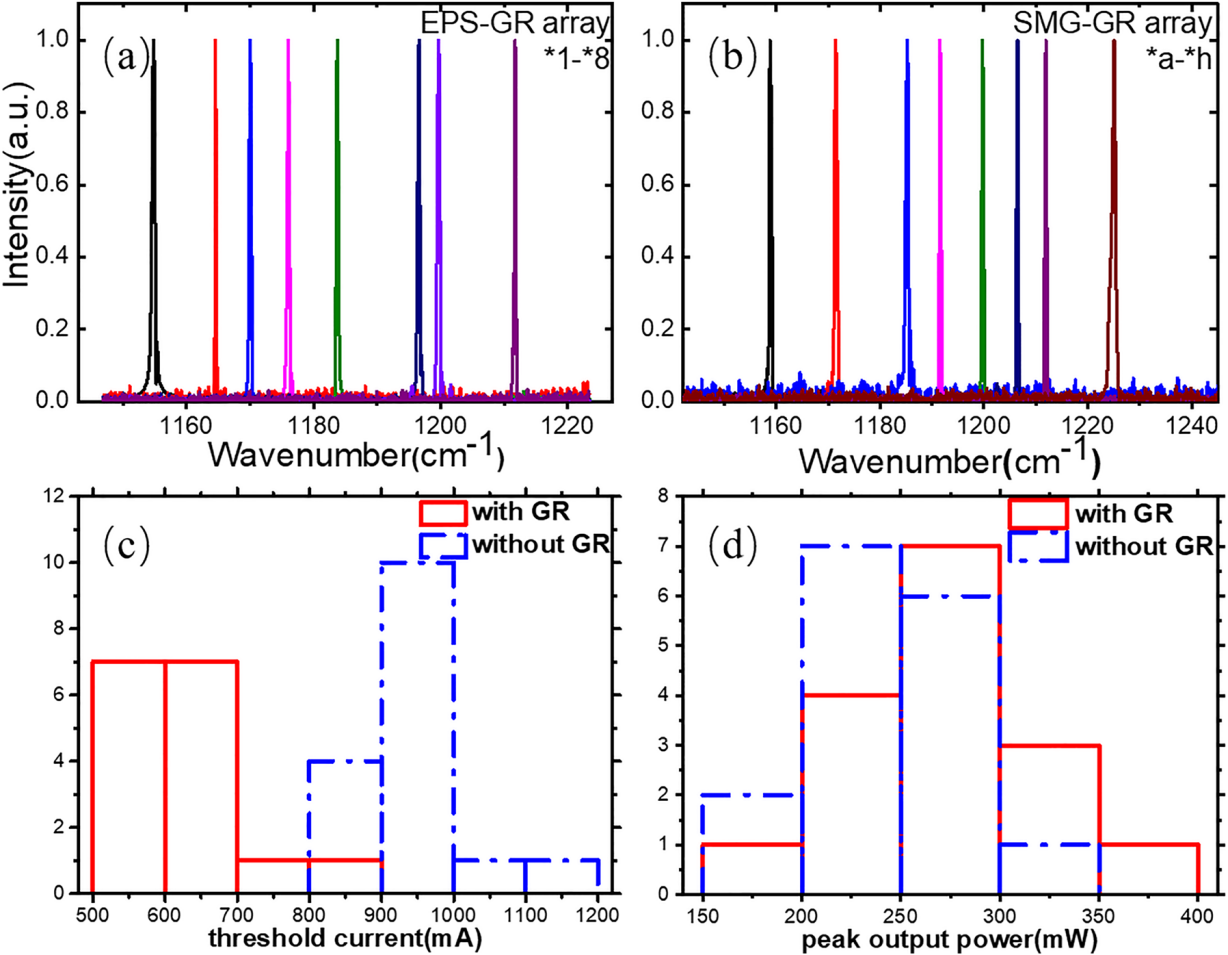
Spectra, threshold current and peak output power of lasers in EPS-GR and SMG-GR arrays. (a) The measured spectra of the *1–*8 in the EPS-GR QCL arrays under 1.2*I*_th_ at a duty cycle of 1% (20 kHz, 0.5 μs) at RT. (b) The measured spectra of the *a–*h in the SMG-GR QCL arrays under 1.2*I*_th_ at a duty cycle of 1% (20 kHz, 0.5 μs) at RT. (c) The threshold current of the 16 lasers in EPS-GR and SMG-GR QCL arrays with and without GR section working. (d) The threshold current of the 16 lasers in EPS-GR and SMG-GR QCL arrays with and without GR section working.

Both EPS-GR and SMG-GR structures were further extended to QCL arrays by introducing semi-insulated InP (Fe). The EPS-GR and SMG-GR QCL arrays both contain 8 channels (*1–*8 and *a–*h) covering a range from 1154 to 1212 cm^−1^ and 1158 to 1225 cm^−1^, corresponding to 8.25–8.67 μm and 8.16–8.63 μm, respectively. The lasing spectra were measured under 1.2 times of threshold current (I_1_ = 1.2*I*_th_) at a duty cycle of 1% (20 kHz, 0.5 μs) at room temperature (RT), as depicted in Figure 8(a) and (b), every laser in the EPS-GR and SMG-GR arrays exhibiting considerable single-mode performance. Figure 8(c) and (d) records the threshold current and peak output power of the total 16 lasers in the EPS-GR and SMG-GR QCL arrays lasing with and without GR section working. It indicates that threshold current of lasers working with GR section is averagely 330 mA lower than those without GR section, mostly gathering from 500 to 700 mA, corresponding to 1.17–1.65 kA/cm^2^. As such, the average peak output power, measured by calibrated thermopile detector, of the lasers in arrays with GR section is approximately 28 mW higher than that without GR section, reaching 272 mW. The improvement in threshold current and peak output power both stresses the importance of the GR section.

**Figure 9:**
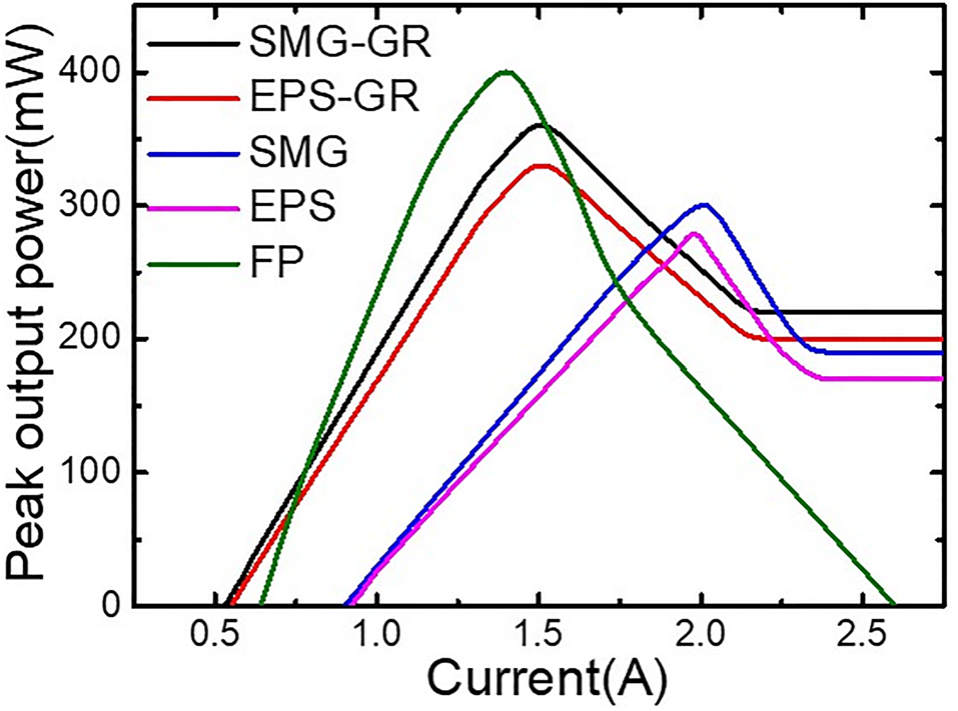
*P*–*I* characteristics of lasers in the SMG-GR, EPS-GR, SMG, EPS and FP arrays at the duty cycle of 1% at RT.

The output power in the arrays was measured with a calibrated thermopile detector placed directly in front of the laser facet at RT. Figure 9 shows the power–current (*P*–*I*) characteristics of lasers in the arrays of the SMG-GR, EPS-GR, SMG without GR working, EPS without GR working and conventional Fabry–Pérot (FP) cavity at a duty cycle of 1% (20 kHz, 0.5 μs). For lasers in the SMG-GR and EPS-GR arrays, *I*_2_ was constantly set at 0.1 A to compensate the loss in the GR section. Noteworthy, the introduction of semi-insulated InP (Fe) contribute to remarkable improvement in output power of lasers in SMG-GR and EPS-GR arrays, both achieving over 300 mW, and have the lowest threshold currents of 0.54 and 0.56 A, respectively. Due to its light extraction near the front facet, the SMG-GR QCL also has the highest peak output power of 360 mW except for the FP cavity, which cannot work in single-mode conditions. Furthermore, the outputs of all types of QCLs other than the FP cavity do not significantly deteriorate after they reach the peak current *I*_1_ and remain almost constant when *I*_1_ > 2.3 A. In contrast, the peak output power of the FP cavity sharply decreases when *I*_1_ > 1.4 A, and the cavity cannot work when *I*_1_ > 2.6 A. This distinctly indicates that the SMG-GR and EPS-GR structures not only guarantee considerable output power but also have a wide dynamic range, allowing the lasers to work at extremely high currents (*I*_1_ > 10 A). These power–current characteristics is speculated due to the leakage through isolated channel after *I*_1_ increases to certain value, namely, the excess current can be regarded as invalid, which instead provides constant power output. The wall-plug efficiencies of SMG-GR and EPS-GR at maximum output are 2.3% and 2.1%, respectively. The favorable output characteristic can be used to manufacture a constant output light source for applications such as high-resolution spectroscopy and also ignore the adverse effects of static electricity on the QCLs.

## 4 Conclusions

We designed and fabricated *λ*∕4 EPS-GR and SMG-GR QCLs to work in stable single-mode condition. The spectra of the EPS-GR QCLs do not unexpectedly broaden or exhibit mode hopping even under extremely large injection currents and pulse widths. The SMSR of the SMG-GR QCLs is 31.4 dB when *I*_1_ = 1.2 A and *τ* = 0.5 μs and is maintained at 24.5 dB when *I*_1_ = 5.0 A and *τ* = 5 μs. The output powers of these two structures are considerably high even near the edge of the gain spectrum, reaching 78 and 124 mW, respectively, due to the optimized coupling strength and light field distribution in the cavity. The devices have swift tuning ability, with a current tuning coefficient Δ*ν*/Δ*I* = −4.82 cm^−1^ A^−1^. In addition, we fabricated the arrays of the *λ*∕4 EPS-GR and SMG-GR QCLs ranging from 8.25 to 8.67 μm and 8.16–8.63 μm in the same manner, respectively. The threshold current of decrease to 0.56 and 0.54 A and the peak output power further increase to 330 and 360 mW for the lasers in the *λ*∕4 EPS-GR and SMG-GR arrays mainly due to the introduction of semi-insulated InP (Fe). Evidently, *λ*∕4 EPS-GR and SMG-GR QCLs are very promising for applications that require single-mode stability and high output, such as gas sensing, industry monitoring and high-resolution spectroscopy. In future work, improvement of the heat dissipation and achievement of CW mode operation will be realized by employing high performance heat sink.
